# Distinction of cardiometabolic profiles among people ≥75 years with type 2 diabetes: a latent profile analysis

**DOI:** 10.1186/s12902-019-0411-2

**Published:** 2019-08-05

**Authors:** Antoine CHRISTIAENS, Michel P. HERMANS, Benoit BOLAND, Séverine HENRARD

**Affiliations:** 1 Fonds national de la recherche scientifique – F.R.S-FNRS, Brussels, Belgium; 2 Clinical Pharmacy Research Group, Louvain Drug Research Institute (LDRI), UCLouvain, Brussels, Belgium; 3 Institute of Health and Society (IRSS), UCLouvain, Brussels, Belgium; 4 Institute of Experimental and Clinical Research, UCLouvain, Brussels, Belgium; 5 Endocrinology unit, Saint-Luc University Hospital, Brussels, Belgium; 6 Geriatric medicine unit, Saint-Luc University Hospital, Brussels, Belgium

**Keywords:** Cardiometabolic profile, Homeostasis model assessment, Older patients, Type 2 diabetes, Type 2 diabetes classification, Type 2 diabetes management

## Abstract

**Background:**

Older patients with type 2 diabetes mellitus represent a heterogeneous group in terms of metabolic profile. It makes glucose-lowering-therapy (GLT) complex to manage, as it needs to be individualised according to the patient profile. This study aimed to identify and characterize subgroups existing among older patients with diabetes.

**Methods:**

Retrospective observational cohort study of outpatients followed in a Belgian diabetes clinic. Included participants were all aged ≥75 years, diagnosed with type 2 diabetes, Caucasian, and had a Homeostasis Model Assessment (HOMA2). A latent profile analysis was conducted to classify patients using the age at diabetes diagnosis and HOMA2 variables, i.e. insulin sensitivity (*HOMA2%-S*), beta-cell-function (*HOMA2%-β*), and the product between both (*HOMA2%-βxS*; as a measure of residual beta-cell function). GLT was expressed in defined daily dose (DDD).

**Results:**

In total, 147 patients were included (median age: 80 years; 37.4% women; median age at diabetes diagnostic: 62 years). The resulting model classified patients into 6 distinct cardiometabolic profiles. Patients in profiles 1 and 2 had an older age at diabetes diagnosis (median: 68 years) and a lesser decrease in *HOMA2%-S,* as compared to other profiles. They also presented with the highest *HOMA2%-βxS* values. Patients in profiles 3, 4 and 5 had a moderate decrease in *HOMA2%-βxS*. Patients in profile 6 had the largest decrease in *HOMA2%-β* and *HOMA2%-βxS*. This classification was associated with significant differences in terms of HbA1c values and GLT total DDD between profiles. Thus, patients in profiles 1 and 2 presented with the lowest HbA1c values (median: 6.5%) though they received the lightest GLT (median GLT DDD: 0.75). Patients in profiles 3 to 5 presented with intermediate values of HbA1c (median: 7.3% and GLT DDD (median: 1.31). Finally, patients in profile 6 had the highest HbA1c values (median: 8.4%) despite receiving the highest GLT DDD (median: 2.28). Other metabolic differences were found between profiles.

**Conclusions:**

This study identified 6 groups among patients ≥75 years with type 2 diabetes by latent profile analysis, based on age at diabetes diagnosis, insulin sensitivity, absolute and residual β-cell function. Intensity and choice of GLT should be adapted on this basis in addition to other existing recommendations for treatment individualisation.

**Electronic supplementary material:**

The online version of this article (10.1186/s12902-019-0411-2) contains supplementary material, which is available to authorized users.

## Background

Type 2 diabetes is one of the most prevalent chronic diseases worldwide, especially among older people aged ≥75 years, in whom prevalence reached 20% in 2017, and is poised to increase over the coming decades [1]. In Europe, the cost per patient per year with diabetes mellitus was estimated at US Dollar 3,100 in 2017. Moreover, diabetes was responsible for 10% of total health care expenditure in 2010 [2]. Diabetes in older patients has therefore a major impact on healthcare systems.

Current classification of diabetes mellitus considers 4 different categories: type 1 diabetes, type 2 diabetes, gestational diabetes and specific rare types of diabetes [3]. In older age, type 2 diabetes is reported to represent 85–90% of all-cause diabetes, ahead of type 1 diabetes, which includes latent autoimmune diabetes in adults [4, 5].

Type 2 diabetes induces specific acute or chronic complications (e.g. microvascular complications from chronic hyperglycaemia) and increases the incident risk of macrovascular complications from various cardiometabolic abnormalities promoting the occurrence of atherosclerosis [6]. These vascular complications promote and intensify the development of several geriatric syndromes in older patients, such as falls, polymedication, cognitive disorders or sensorial disorders [6, 7]. The aim of glucose lowering therapy (GLT) in these patients is to control hyperglycaemia and its associated morbidity and mortality. Nevertheless, in older patients with type 2 diabetes, GLT should be adapted according to patient’s characteristics in order to be intense enough to avoid microvascular complications but light enough to prevent potential side-effects of GLT, mainly hypoglycaemia, as it also increases morbidity and mortality [7]. These considerations offer only a narrow frame to perform a safe and effective GLT management in patients aged 75 years or more with type 2 diabetes. Several recent guidelines provide recommendations about GLT management in older patients with diabetes, in terms of hyperglycaemia, risk factors and complications [8, 9]. These guidelines and other reports all insist on treatments’ individualisation in order to give tailored medication for each patient [8, 10–15]. At present, factors currently considered in this treatment individualisation are related to the type of diabetes [3], but also to prevalent comorbidities, geriatric syndromes, nutrition issues, physical activity, age-specific aspects of pharmacotherapy, ethnic disparities and estimated life expectancy [8, 11].

Indeed, type 2 diabetes is a complex condition with marked heterogeneity in pathophysiological mechanisms leading to hyperglycaemia and cardiometabolic comorbidities between patients. Ageing process enhances this heterogeneity, adding other conditions, such as nutritional deficits, sarcopenia, additional stresses on pancreatic beta-cells and micro-inflammation [16–18].

Yet, current guidelines for older patients do not suggest taking into account characteristics related to the pathophysiology of diabetes or severity of residual beta-cell function (BCF) loss. Therefore, it is of interest to consider these factors in GLT individualisation in order to improve the quality, efficacy and safety of GLT management in older patients.

Therefore, the aim of the present study was to assess the heterogeneity of cardiometabolic features in patients aged 75 years or more with type 2 diabetes and to classify them into relevant cardiometabolic profiles using mixture models as Latent Profile Analysis (LPA).

## Methods

### Study design and patient selection

A retrospective cohort study of outpatients followed by the same investigator (MPH) between 2000 and 2017 and attending a Belgian university diabetes clinic was conducted. Among the 266 Caucasian patients followed in the diabetes clinic and aged ≥75 years at the last two visits to the endocrinologist, 147 participants had a Homeostasis Model Assessment (HOMA2) after the diagnosis of their type 2 diabetes. All 147 participants were GAD-antibodies-negative. Type 2 diabetes was defined according to the Expert Committee on the Diagnosis and Classification of Diabetes Mellitus [3].

This study was approved by the local Ethics Committee (Commission d’Ethique Hospitalo-Facultaire, Cliniques universitaires Saint-Luc, Brussels, Belgium; ref. B403/2017/16NOV/521).

### Data collection

A first part of the data was collected at the time of the HOMA2 assessment. Data included anthropometric (weight, body mass index and fat mass proportion), biochemical (HbA1c) and ongoing GLT (drug molecules and doses).

Body mass index (BMI; kg/m^2^) was calculated as [Weight(kg) × Height(m)^− 2^]. Body fat mass (%) was measured using a BodyFat Analyser (Omron BF 500; Omron Healthcare Europe B.V., Hoofddorp, The Netherlands). HbA1c was expressed in NGSP nomenclature (%) and was converted to IFCC nomenclature (mmol/mol) using the NGSP convertor (www.ngsp.org/convert1.asp).

Insulin sensitivity and beta-cell function were assessed using the computer-based homeostasis model assessment (HOMA2, *http://www.dtu.ox.ac.uk*) [19]. HOMA2 parameters were calculated from triplicates of fasting glucose and insulin level, sampled after a sufficient period of GLT washout (i.e. between 1 to 5 days, according to the molecules involved). Values of insulin secretion (*HOMA2%-β (%)*; normal 100%) were plotted as a function of insulin sensitivity (*HOMA2%-S (%)*; normal 100%), defining a *hyperbolic product area* (*HOMA2%-βxS (%*^*2*^*)*; normal 100%). This product described the interaction between insulin sensitivity and insulin secretion, or more precisely, the true latent beta-cell function (BCF) indexed by insulin sensitivity. It approximates the magnitude of glucose homeostasis deficit and the required GLT intensity [20].

GLT data corresponded to the treatment taken one week before the HOMA2 realization. Drugs were transcribed into Anatomical Therapeutic Chemical (ATC) codes and grouped by GLT classes (A10A-Insulin, A10BA-biguanides, A10BB-sulfonylureas, A10BF-alpha-glucosidase-inhibitors, A10BG-thiazolidinediones, A10BH-DPP4-inhibitors, A10BJ-GLP1-receptor agonists, A10BX-other). Sulfonylureas and repaglinide were considered as “*Oral hypoglycaemic agents (OHA)”* and insulin and OHA were considered as “*Hypoglycaemic agents (HA)”.* Patients with no GLT were considered as “*Lifestyle changes only*”. Treatment doses were collected and converted into Defined Daily Dose (DDD), according to the ATC/DDD Index 2018 [21]. For each patient, a sum of the GLT drugs doses, expressed in DDD, was computed and described hereafter as “*GLT’s total doses”*.

The second part of the data was collected at the time of the last consultation at the diabetes clinic, at which all patients were ≥ 75 years, and included socio-demographic (age, sex) and diabetes-related data (age at diabetes diagnosis, comorbidities, vascular complications). Micro-angiopathic complications were defined as: neuropathy (clinical examination of knee and ankle reflexes; Semmes-Weinstein monofilament test, confirmed by lower-limbs electromyography) and diabetic retinopathy (based on retinal examination by an experienced ophthalmologist and/or fluorescein angiography). Diabetic nephropathy was not taken into account in this study because of its high prevalence in older age and its multiple aetiologies that cannot be attributed de facto to chronic hyperglycaemia.

Macro-angiopathic complications included coronary artery disease (CAD: myocardial infarction, angioplasty, stenting, revascularization surgery and/or significant coronary stenosis confirmed by angiography), cerebrovascular disease (CVD) or peripheral artery disease (PAD). CVD was defined according to *UK Prospective Diabetes Study* criteria: any neurological deficit ≥1 month, without distinction between ischemic, embolic and haemorrhagic events [22]. PAD was diagnosed from medical history of lower-limb claudication; clinical or imaging evidence for ischemic diabetic foot; history of angioplasty, stenting, revascularization surgery; and/or lower-limb artery stenosis at Doppler ultrasonography or angiography.

### Statistical analysis

Continuous data were expressed as medians (P25, P75). Categorical data were expressed as number of people and percentages. Continuous variables were compared between 2 groups using Mann Whitney test, and between ≥3 groups using Kruskal-Wallis test. Categorical variables were compared between groups using Pearson’s χ^2^ test, Pearson’s χ^2^ test with Yates correction, Fisher’s exact test or Fisher Freeman Halton’s test, according to the conditions of validity of each test.

In order to identify profiles of patients with type 2 diabetes a latent profile analysis (LPA) was performed using the following continuous discriminant variables (indicators): insulin sensitivity (*HOMA2%-S*), BCF (*HOMA2%-β*), hyperbolic product *βxS* (*HOMA2%-βxS*) and age at diabetes diagnosis. Models with 2 to 7 profiles were ran. Evaluative information was used to select the best model, e.g. the model with the lowest Akaike information criteria, Bayesian Information Criterion (BIC) and Log Likelihood (LL) [23]. In addition, the likelihood ratio test was used to compare a model with k-1 profiles with a model with k profiles. Finally, posterior probabilities, i.e. the probability of each patient of belonging to each profile, were computed for the final selected model. An average posterior probability per group ≥0.70 was used to consider whether profiles were sufficiently separated from each other.

Statistical analyses were performed using IBM SPSS Statistics 25® software or R software (R × 64 version 3.4.1). A *p*-value< 0.05 was considered statistically significant.

## Results

### Patients’ characteristics

The 147 older patients (≥ 75 years of age; 37% women) had a median age of 62.0 years at diabetes diagnosis and a median duration of diabetes of 19.0 years at the last visit at the diabetes clinic (Table 1, left column). According to HOMA2-modeling, median insulin sensitivity was 47.4% and median BCF was 49.3%. Median hyperbolic product of insulin sensitivity and beta-cell function *βxS*) was 25.0% and median HbA1c was 7.1% (54 mmol/mol) at the time of the HOMA2 testing.

**Table 1:** Patients’ characteristics by cardiometabolic profiles created in latent profile analysis (*N* = 147)

Variables	Total (*N* = 147) Median [P25; P75] *or* n (%)	Profile 1 (*n* = 16) Median [P25; P75] *or* n (%)	Profile 2 (*n* = 14) Median [P25; P75] *or* n (%)	Profile 3 (*n* = 23) Median [P25; P75] *or* n (%)	Profile 4 (*n* = 29) Median [P25; P75] *or* n (%)	Profile 5 (*n* = 28) Median [P25; P75] *or* n (%)	Profile 6 (n = 37) Median [P25; P75] *or* n (%)	*P*-value
Characteristics at the time of the last consultation								
Age, *in years*	80.0 [77.0; 83.0]	80.5 [76.0; 82.8]	80.0 [78.0; 84.0]	77.0 [76.0; 81.0]	79.0 [76.0; 82.0]	80.0 [77.0; 83.8]	81.0 [77.0; 84.0]	0.545
Women	55 (37.4)	6 (37.5)	4 (28.6)	12 (52.2)	14 (48.3)	7 (25.0)	12 (32.4)	0.278
Family history of diabetes*	52 (35.4)	4 (25.0)	4 (30.8)	5 (21.7)	11 (37.9)	11 (39.3)	17 (49.6)	0.348
Diabetes duration, *in years*	19.0 [12.0; 27.0]	12.0 [3.3; 14.8]	15.0 [9.8; 20.3]	12.0 [5.0; 23.0]	20.0 [15.5; 28.0]	23.5 [17.3; 29.0]	22.0 [14.5; 27.5]	< 0.001
Characteristics at the time of HOMA2								
Age at HOMA2*, in years*	72.0 [69.0; 76.0]	73.5 [70.3; 76.5]	72.0 [70.0; 73.3]	71.0 [64.0; 76.0]	71.0 [66.0; 77.0]	71.0 [68.3; 74.8]	73.0 [68.0; 77.0]	0.714
BMI, *in kg/m*^*2*^	28.3 [25.7; 31.2]	26.8 [24.9; 29.6]	26.2 [24.7; 33.1]	28.0 [25.8; 33.3]	29.6 [26.6; 34.6]	27.9 [25.7; 29.6]	28.2 [25.5; 31.7]	0.037
BMI [18–25[kg/m^2^	33 (22.4)	4 (25.0)	5 (35.7)	3 (13.0)	8 (27.6)	9 (32.1)	4 (10.8)	
BMI [25–30[kg/m^2^	68 (46.3)	10 (62.5)	6 (42.9)	9 (39.1)	8 (27.6)	14 (50.0)	21 (56.8)	
BMI ≥30 kg/m^2^	45 (30.6)	2 (12.5)	3 (21.4)	11 (47.8)	13 (44.8)	4 (14.3)	12 (32.4)	
HbA1c†, *in %*	7.1 [6.4; 8.2]	6.6 [6.0; 7.9]	5.4 [5.2; 6.6]	7.0 [6.4; 7.9]	7.4 [7.1; 8.0]	6.8 [5.8; 7.2]	8.4 [7.8; 9.1]	< 0.001
Indicators used in latent profile analysis								
Age at diagnosis, *in years*	62.0 [54.0; 70.0]	70.5 [63.3; 74.0]	68.0 [61.0; 70.5]	64.0 [58.0; 74.0]	58.0 [54.5; 62.0]	55.5 [47.8; 67.8]	61.0 [52.0; 65.0]	< 0.001‡
HOMA2%-S, *in %*	47.4 [32.1; 73.0]	50.5 [47.0; 56.4]	84.1 [58.2; 115.8]	22.3 [15.1; 31.4]	35.2 [29.1; 42.9]	86.3 [74.1; 106.9]	48.3 [34.3; 40.2]	< 0.001‡
HOMA2%-β, in %	49.3 [32.4; 72.6]	71.6 [68.1; 76.1]	66.5 [47.6; 111.1]	111.6 [95.1; 135.5]	56.5 [46.8; 65.5]	39.2 [32.0; 47.9]	27.3 [21.7; 34.9]	< 0.001‡
HOMA2%-βxS*, in %*	25.0 [16.0; 37.0]	36.0 [33.3; 40.5]	56.0 [52.8; 64.3]	28.0 [17.0; 34.0]	20.0 [17.0; 24.0]	34.5 [28.3; 39.8]	13.0 [9.0; 16.0]	< 0.001‡

HOMA2%-S: insulin sensitivity assessed by Homeostasis Model Assessment 2 (HOMA2); HOMA2%-β: beta-cell function assessed by HOMA2; HOMA2%-βxS: Hyperbolic product between beta-cell function and insulin sensitivity. BMI: body mass index (kg.m^−2^).* 3 missing values (2.0%). † 1 missing value (0.7%). ‡ Differences were expected as these indicators were included in the LPA to create the 6 profiles

### Profiles of older patients with type 2 diabetes

Using latent profile analysis, a 6-profile model was the best-fitting model based on evaluative information (see Additional file 1). In addition, in this model, the average probability of each patient to belong to each group ranged from 0.904 in profile 4 to 0.977 in profile 2, showing good separation between profiles (see Additional file 2).

### Profiles’ characteristics collected at the HOMA2 assessment

**Fig. 1:**
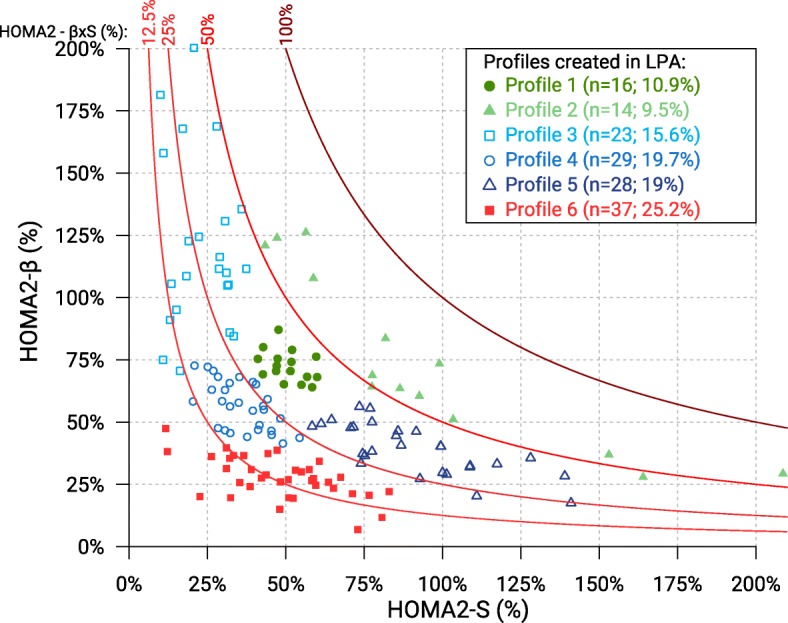
Distribution of older patients with type 2 diabetes on HOMA2 graph, labeled according to the 6 profiles obtained from the Latent Profile Analysis (LPA). This graph represents insulin sensitivity (*HOMA2%-S*) on the x-axis and beta-cell function (*HOMA2%-β*) on the y-axis, both calculated by Homeostasis Model Assessment (HOMA2). The product of *HOMA2%-S* and *HOMA2%-β* is represented on the hyperbolic axis (*HOMA2%-βxS*) at four levels (100, 50, 25, 12.5%)

**Fig. 2:**
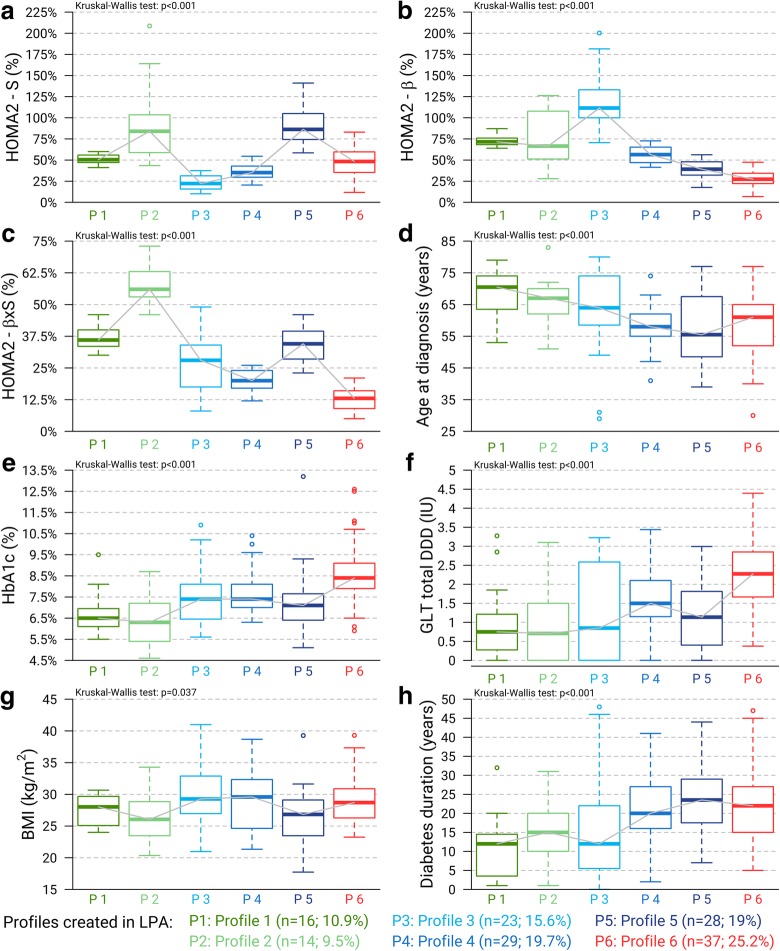
Distribution of patients’ diabetes characteristics according to the 6 profiles created in latent profile analysis. Boxplot of patients’ (**a**) insulin sensitivity (*HOMA2%-S*) calculated by Homeostasis Model Assessment (HOMA2), (**b**) beta-cell function (*HOMA2%-β*) calculated by Homeostasis Model Assessment (HOMA2), (**c**) hyperbolic product of insulin sensitivity and beta-cell function (*HOMA2%-βxS*), (**d**) Age at diagnosis of type 2 diabetes mellitus, (**e**) HbA1c collected at the time of HOMA2 assessment, (**f**) Glucose-lowering therapy (GLT) total dose, used just before the HOMA2 assessment, and expressed in Defined Daily Dose (one unrepresented outlier patient in profile 6 whose GLT total DDD = 6.88), (**g**) BMI (kg/m^2^; one unrepresented outlier patient in profile 3 whose BMI = 58.56 kg/m^2^), and (**h**) duration of diabetes until the last endocrinology consultation, according to the 6 profiles created by the Latent Profile Analysis (LPA). Statistical comparisons between profiles were performed using a Kruskal-Wallis test

HOMA2 was realized at similar median ages in the 6 profiles, between 71.0 and 73.5 years (*p* = 0.714) (Table 1). All participants’ values were plotted on a HOMA2 graph presenting the relationship between *HOMA2%-β* and *HOMA2%-S* (Fig. 1). Each profile of patients was distinctly delimited in terms of *HOMA2%-β*, *HOMA2%-S*. *HOMA2%-βxS* values were different (*p* < 0.001) across the six profiles (Table 1; Fig. 2c).

No significant difference was found in the six profiles in terms of sex ratio (*p* = 0.278).

Patients in profiles 1 (*n* = 16; 10.9%) and 2 (*n* = 14; 9.5%) had an older age at diabetes diagnosis (median: 70.5 years and 68.0 years, respectively) and had a slight decrease in *HOMA2%-βxS* (median: 36.0 and 56.0%, respectively) (Table 1; Fig. 2c). Patients in profile 2 had a higher insulin sensitivity than patients in profile 1 (Table 1; Fig. 2a). As profile 2 also had preserved *beta-secretion (66.*5%), its *HOMA2%-βxS* was the highest (56%). From profiles 3 (*n* = 14) to 5 (*n* = 29), insulin sensitivity increased, and beta-cell function decreased inversely, resulting in a moderate decrease in *HOMA2%-βxS* in all 3 profiles (median: 28.0, 20.0 and 34.5%, respectively; Table 1; Fig. 2c). Profile 6 (*n* = 37) had the lowest beta-cell function (27.3%) and thereby the lowest *HOMA2%-βxS* (median: 13.0%) (Table 1; Fig. 2c).

**Fig. 3:**
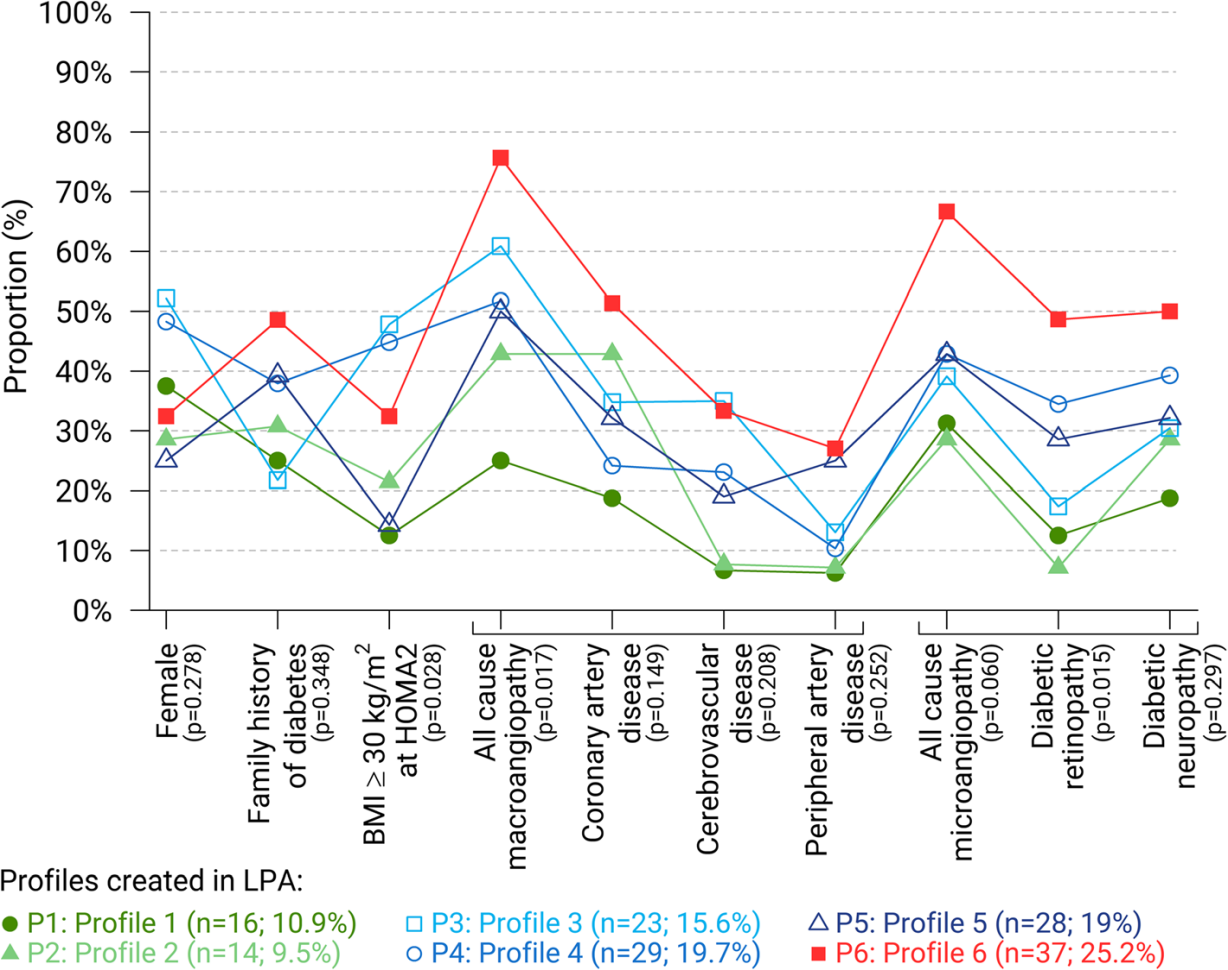
Prevalence of women, family history of diabetes, BMI ≥30 kg/m^2^, all cause macroangiopathy (coronary artery disease, cerebro-vascular disease and peripheral artery disease) and all cause microangiopathy (diabetic retinopathy and diabetic neuropathy) in each of the 6 profiles created by LPA. Statistical comparisons between profiles were performed using Pearson’s χ^2^ test, Pearson’s χ^2^ test with Yates correction or Fisher Freeman Halton’s test according to the conditions of validity of each test

The six profiles were also significantly different in terms of BMI (*p* = 0.037). Profiles 1 and 2 had the lowest median BMI, while profile 3 and 6 had the highest median values. Obesity (i.e. BMI ≥ 30.0 kg/m^2^) was less prevalent in profiles 1 (12.5%), 2 (21.4%) and 5 (14.3%) than in profiles 3 (47.8%), 4 (44.8%) and 6 (32.4%) (*p* = 0.028). There was no significant difference in fat mass proportion between profiles (*p* = 0.137), nor in abdominal circumference (*p* = 0.129) (Table 1; Fig. 2; Fig. 3). Finally, the median HbA1c value was higher in profile 6 than in profiles 1, 2, 3 and 5 (*p* < 0.001) (Table 1).

Regarding the use of GLT in the six profiles, significant difference was observed in terms of number of glucose-lowering agents (*p* < 0.001) (Table 2). Profiles 1, 2, 3 and 5 had lower number of GLTs than profile 6. Moreover, a higher prevalence of GLT-bi- and -tri-therapy was found in profile 6. There were no differences in the proportions of patients receiving biguanides, except in profiles 2 and 3 (those with higher residual beta-secretion) (Fig. 1). Insulin was prescribed more frequently in profiles 2, 4 and 6 than in other profiles, as was prescription of hypoglycaemic agents or oral hypoglycaemic agents (Table 2).

**Table 2:** Patients’ glucose-lowering therapy at the time of HOMA2 by pathophysiologic profiles (*N* = 147)

Variable	Total (*N* = 147) Median [P25; P75] or n (%)	Profile 1 (*n* = 16) Median [P25; P75] *or* n (%)	Profile 2 (*n* = 14) Median [P25; P75] *or* n (%)	Profile 3 (*n* = 23) Median [P25; P75] *or* n (%)	Profile 4 (*n* = 29) Median [P25; P75] *or* n (%)	Profile 5 (*n* = 28) Median [P25; P75] *or* n (%)	Profile 6 (*n* = 37) Median [P25; P75] *or* n (%)
Number of GLT drugs*	2.00 [1.00; 2.00]	1.00 [1.00; 1.75]	1.00 [0.00; 2.00]	1.00 [0.00; 2.00]	2.00 [1.00; 2.00]	1.00 [1.00; 2.00]	2.00 [2.00; 2.00]
Lifestyle changes only	21 (14.3)	3 (18.8)	4 (28.6)	7 (30.4)	1 (3.4)	6 (21.4)	0 (0.0)
Monotherapy	52 (35.4)	9 (56.3)	5 (35.7)	8 (34.8)	12 (41.4)	11 (39.3)	7 (18.9)
Bitherapy	61 (41.5)	3 (18.8)	5 (35.7)	6 (26.1)	13 (44.8)	11 (39.3)	23 (62.2)
Tritherapy	13 (8.8)	1 (6.3)	0 (0.0)	2 (8.7)	3 (10.3)	0 (0.0)	7 (18.9)
GLT’s total dose, in DDD	1.50 [0.75; 2.28]	0.75 [0.35; 1.11]	0.71 [0.11; 1.33]	0.85 [0.00; 2.59]	1.50 [1.15; 2.10]	1.14 [0.41; 1.79]	2.28 [1.67; 2.85]
GLT’s total dose among users, in DDD	1.67 [0.89; 2.58]	0.85 [0.50; 1.43]	0.79 [0.69; 1.69]	1.81 [0.83; 2.86]	1.53 [1.21; 2.11]	1.52 [0.85; 2.07]	2.28 [1.67; 2.85]
GLT classes							
Biguanides							
Use	79 (53.7)	8 (50.0)	5 (35.7)	9 (39.1)	18 (62.1)	16 (57.1)	23 (62.2)
DDD among users	0.85 [0.57; 1.28]	0.68 [0.48; 0.85]	0.50 [0.43; 0.50]	0.85 [0.85; 1.28]	0.85 [0.50; 1.17]	0.85 [0.85; 1.28]	0.85 [0.80; 1.28]
Insulin							
Use	29 (19.7)	1 (6.3)	4 (28.6)	3 (13.0)	11 (37.9)	0 (0.0)	10 (27.0)
DDD among users	1.00 [0.60; 1.35]	0.75	0.34 [0.31; 0.41]	1.95 [1.40; 2.16]	1.05 [0.73; 1.24]	NA	1.28 [0.93; 1.46]
OHA							
Use	79 (53.7)	8 (50.0)	5 (35.7)	10 (43.5)	15 (51.7)	12 (42.9)	29 (78.4)
DDD among users	1.50 [1.00; 1.89]	1.00 [0.94; 1.00]	1.43 [1.00; 1.50]	1.00 [0.56; 2.04]	1.50 [0.88; 1.50]	1.21 [0.94; 1.57]	1.50 [1.43; 2.00]
Hypoglycemic agents							
Use	101 (68.7)	9 (56.3)	6 (42.9)	13 (56.5)	24 (82.8)	12 (42.9)	37 (100.0)
DDD among users	1.43 [1.00; 1.79]	1.00 [0.75; 1.00]	1.38 [0.81; 1.77]	1.00 [0.75; 2.14]	1.24 [0.97; 1.50]	1.21 [0.94; 1.57]	1.50 [1.35; 2.00]

*GLT* glucose-lowering therapy, *DDD* defined daily dose, *OHA* Oral hypoglycemic agents (=Sulfonylureas or Glinides); Hypoglycemic agents: Insulin and/or OHA. * Statistically significant difference between groups (*p*-value < 0.001). *NA* not applicable

In addition to differences within profiles in GLT agents, the median GLT total doses, expressed as daily defined doses (DDD), were different between profiles (*p* < 0.001). Profile 1 and 2 had the lowest median DDD, profiles 3 to 5 had intermediate median DDD and profile 6 had the highest median DDD (Table 2). Profile 6 was significantly different in that respect from profiles 1, 2, 3 and 5 (*p* < 0.001).

### Diabetes complications and comorbidities of each profile at the time of the last consultation

At the date of the last consultation to the diabetes clinic, profiles 1 and 2 had the lowest prevalence of diabetic retinopathy (13 and 7%, respectively), and profile 6 the highest prevalence (68.6%) (*p* = 0.015; Table 3; Fig. 3). Profiles 1 and 2 also had the lowest prevalence of diabetic neuropathy and all-cause-microangiopathy (diabetic retinopathy and neuropathy), and profile 6 the highest one, without statistically significant differences (Table 3; Fig. 3).

**Table 3:** Diabetes complications and comorbidities according to subgroups created from the latent profile analysis (*N* = 147)

Variable	Total (*N* = 147) n (%)	Group 1 (*n* = 16) n (%)	Group 2 (*n* = 14) n (%)	Group 3 (*n* = 23) n (%)	Group 4 (*n* = 29) n (%)	Group 5 (*n* = 28) n (%)	Group 6 (*n* = 37) n (%)	*P*-value
Nephropathy	97 (66.0)	13 (81.3)	7 (50.0)	15 (65.2)	20 (69.0)	17 (60.7)	25 (67.6)	0.584
Microangiopathy	68 (46.3)	5 (31.3)	4 (28.6)	9 (39.1)	13 (44.8)	12 (42.9)	25 (67.6)	0.060
Diabetic retinopathy	43 (29.3)	2 (12.5)	1 (7.1)	4 (17.4)	10 (34.5)	8 (28.6)	18 (68.6)	0.015
Diabetic neuropathy	52 (35.9)	3 (18.8)	4 (28.6)	7 (30.4)	11 (39.3)	9 (32.1)	18 (50.0)	0.297
Macroangiopathy	81 (55.1)	4 (25.0)	6 (42.9)	14 (60.9)	15 (51.7)	14 (50.0)	28 (75.7)	0.017
Coronary artery disease	52 (35.4)	3 (18.8)	6 (42.9)	8 (34.8)	7 (24.1)	9 (32.1)	19 (51.4)	0.149
Cerebro-vascular disease	28 (23.0)	1 (6.7)	1 (7.7)	7 (35.0)	6 (23.1)	4 (19.0)	9 (33.3)	0.208
Peripheral artery disease	25 (17.0)	1 (6.3)	1 (7.1)	3 (13.0)	3 (10.3)	7 (25.0)	10 (27.0)	0.252

Microangiopathy = diabetic retinopathy and/or diabetic neuropathy; Macroangiopathy = coronary artery disease and/or cerebrovascular disease and/or peripheral artery disease

Differences were also found in terms of all-cause macroangiopathy prevalence (*p* = 0.017). Profile 6 had the highest prevalence of macroangiopathy (75.7%), unlike profile 1 (25.0%). Profiles 2 to 5 had intermediate prevalence of macroangiopathy (42.9, 60.9, 51.7 and 50.0% respectively). Finally, a familial history of type 2 diabetes was less prevalent in profile 3 (21.7%) and profile 1 (25.0%) than in other profiles, in particular profile 6 (48.6%), but no significant difference was found between profiles (*p* = 0.348). Among the 6 profiles, no significant difference was found in terms of age at the time of last consultation (*p* = 0.545) (Table 1; Fig. 3; Table 3).

## Discussion

The aim of the present study was to classify older patients with type 2 diabetes into profiles using a LPA methodology based on their metabolic features, in order to select more appropriate GLT in terms of their diabetes attributes and metabolic phenotype, and doing so to add another dimension to treatment individualisation [8] based on diabetes characteristics.

The indicators used as discriminant variables input for LPA were selected on the basis of recent literature. First, as suggested in several studies, age at diabetes’ diagnosis is a major determinant of metabolic differences. Cardio-metabolic profile is usually less severe in patients with an older age at diabetes diagnosis than in those who are diagnosed younger. The former have lower HbA1c, fasting plasma glucose, fasting insulin, insulin resistance, triglyceride levels, LDL-cholesterol, BMI, obesity prevalence and family history of diabetes [24–26]. Patients diagnosed with diabetes at an older age also have a lower risk of developing diabetic retinopathy, regardless of known diabetes duration [27]. This suggests that their diabetes might have a lower propensity of generating microvascular complications.

Furthermore, *HOMA2%-S* and *HOMA2%-β* were used in order to distinct patients in terms of intrinsic glucose homeostasis characteristics, allowing to better select among GLT alternatives. One advantageous feature of our model is to have *HOMA2%-βxS* among input variables, bringing essential information on residual BCF to better identify patients whose needs and intensity of GLT escalation are more marked [19].

The use of these indicators allowed classifying patients into six distinct profiles. It highlights important phenotypic differences across patients sharing a common and seemingly unambiguous diagnosis of type 2 diabetes. Firstly, patients of profiles 1 and 2 had both the highest age at diabetes diagnosis combined with the highest *βxS*, whereas profile 6 patients had the youngest age at diabetes diagnosis and the lowest *βxS*. A link seems to exist between age at diabetes diagnosis and magnitude of glucose homeostasis’ impairment, as shown in previous studies [24, 25]. This also implies that patients with an older age at diabetes diagnosis may need less intensive GLT, in terms of dose and drug of choice (e.g. use of hypoglycaemic agent). Inappropriate prescribing of hypoglycaemic agents in patients with late-onset type 2 diabetes may induce severe hypoglycaemic events.

Secondly, cardiometabolic risk, as shown by indices of insulin resistance, macrovascular comorbidities and BMI was very different between profiles. Some patients’ profiles had lower BMI, lower insulin resistance and few macrovascular complications (e.g. profile 1), while other profiles had higher values of these variables (e.g. profile 6). Profiling older patients with type 2 diabetes thus confirms the rationale of bringing under control modifiable risk factors taking into account the cardiometabolic risk profile for the corresponding profile of individual patients.

The LPA method used allowed for distinguishing patients based on degree of insulin resistance and/or BCF loss. The quantification of these variables provides useful information to individualise GLT (e.g. hypoglycaemic agents when impaired BCF is the major driver of hyperglycaemia or biguanides when insulin resistance is in the foreground). This is all the more relevant given the absence of phenotypic overlap of different profiles of type 2 diabetes in older patients.

The strengths of the present study are twofold. First, all patients were followed by the same endocrinologist and data were prospectively collected by one dedicated clinician. This allows for standardization of all bioclinical measurements, increases as such data’s quality and accuracy. Second, the HOMA2 was based on triplicates of fasting glucose and insulin levels sampled after a sufficient period of GLT washout. However, this sample of patients, most of whom Caucasians from a well-off Brussels suburb, was followed at a single-centre diabetes clinic, and may not de facto be representative of other populations of older patients with type 2 diabetes of various ethnicities.

Recently, Ahlqvist et al. provide a refined classification of diabetes using a data-driven cluster analysis [28], realised on a large cohort of Swedish patients with diabetes (ANDIS cohort, *N* = 8980) and replicated on three independent cohorts (*N* = 5795). It classified patients into five clusters. Despite some similarities in the aims and variables chosen to classify patients, the study of Ahlqvist et al. differed from the present study in many ways.

First, the data used in ANDIS cohort were collected on incident cases at the time of the diabetes diagnosis (median time at inclusion = 40 days after diagnosis) in adult patients aged from 18 to 96 years, with a mean age at diagnosis of 60.2 years. Our study included prevalent cases of patients diagnosed with type 2 diabetes ≥75 years, with a median age at diagnosis of 62.0 years. Then, the inclusion criteria of Ahlqvist et al. were not restricted to type 2 diabetes but included all types of diabetes. The analytical method was a data-driven clustering, a classification method based on different theoretical approach as compared to latent profile analysis used in the present study. Finally, Ahlqvist et al. used six variables classifying patients into subgroups: three were identical to those used in the present study (HOMA2%-β, HOMA2%-S and age at diabetes onset), while two were not used (body mass index (BMI), GAD-antibodies and HbA1c). In the present study, BMI was not used, as it is not an optimal measure for obesity in older patients [29]. GAD-antibodies were not used, as the present study included only patients with type 2 diabetes. Regarding HbA1c, the present study used HOMA2%-βxS instead, assessing the blood glucose control in patients taking glucose lowering therapies.

In the future, it might be of interest to assess the reproducibility of this study by increasing the number of patients, by recruiting older patients with diabetes followed by general practitioners and/or by running a study with a prospective design. It would allow predicting whether patients are ascribed to their appropriate profile and, accordingly, to propose therapeutic recommendations based on the patient’s cardiometabolic profile, keeping in mind that such recommendations could only serve as complements to existing criteria for standards of care individualisation and current guidelines [8].

## Conclusions

In conclusion, our study confirms the heterogeneity of cardiometabolic profiles in older type 2 diabetes patients, generating six profiles by LPA. The characterization of six distinct profiles could serve as decision-support indicators for choosing GLT, combined with existing criteria of therapeutic individualisation for older patients. Such classification could contribute to refine the current decision processes related to the control of hyperglycaemia, while limiting the risk of side effects such as hypoglycaemic episodes or therapeutic failure, aiming at a better overall management of the disease and its complications.

## Additional files

Additional file 1: Latent profile analysis: Model fit statistics. Evaluative information (Goodness-of-fit statistics) for each k-profile model, including Log likelihood, Akaike Information Criterion (AIC), Bayesian Information Criterion (BIC) and Log Likelihood Ratio Test (LLRT). These statistics were used to select the best fitting number of profiles for the final latent profile anlaysis model. (DOCX 14 kb)

Additional file 2: Posterior probabilities associated with each profile in the six-profile model (*N* = 147). Posterior probabilities associated with each profile in the six-profile model (*N* = 147). (DOCX 16 kb)

## Data Availability

The datasets generated and/or analysed during the current study are not publicly available due to restrictions on patients’ anonymity but are available from the corresponding author on reasonable request.

## Abbreviations

ATC: Anatomical Therapeutic Chemical (ATC) Classification System
BCF: Beta Cell Function
BMI: Body Mass Index
DDD: Defined Daily Dose
GLT: Glucose Lowering Therapy
HOMA2: Homeostasis Model Assessment 2
LADA: Latent Autoimmune Diabetes in Adults
LPA: Latent Profile Analysis
OHA: Oral Hypoglycaemic Agents
