# Supplementary material for: Distinction of cardiometabolic profiles among people ≥75 years with type 2 diabetes: a latent profile analysis
Source: BMC Endocr Disord. 2019 Aug 5;19:85. doi: 10.1186/s12902-019-0411-2 (PMC6683451; doi:10.1186/s12902-019-0411-2)
Supplement: Supplementary file 1 — Latent profile analysis: Model fit statistics. Evaluative information (Goodness-of-fit statistics) for each k-profile model, including Log likelihood, Akaike Information Criterion (AIC), Bayesian Information Criterion (BIC) and Log Likelihood Ratio Test (LLRT). These statistics were used to select the best fitting number of profiles for the final latent profile anlaysis model. (DOCX 14 kb) [file 12902_2019_411_MOESM1_ESM.docx]

**ADDITIONAL FILE**

Additional file 1. Latent profile analysis: Model fit statistics.

| Model | Goodness-of-fit statistics | | | |
| --- | --- | --- | --- | --- |
|  | Log Likelihood | AIC | BIC | LLRT p-value* |
| 1-profile | -2616.4 | 5248.8 | 5272.7 |  |
| 2-profile | -2548.7 | 5131.5 | 5182.3 | <0.001 |
| 3-profile | -2503.6 | 5059.1 | 5136.9 | <0.001 |
| 4-profile | -2475.1 | 5020.2 | 5124.9 | <0.001 |
| 5-profile | -2446.4 | 4980.8 | 5112.4 | <0.001 |
| 6-profile | -2416.9 | 4939.8 | 5098.3 | <0.001 |
| 7-profile | -2409.7 | 4943.4 | 5128.8 | 0.111 |

AIC = Akaike Information Criteria; BIC = Bayesian Information Criteria; LLRT = Log Likelihood Ratio Test.

*A p-value <0.05 indicates that a k profile model provides better fit than a k-1 profile model.
