# Supplementary material for: Distinction of cardiometabolic profiles among people ≥75 years with type 2 diabetes: a latent profile analysis
Source: BMC Endocr Disord. 2019 Aug 5;19:85. doi: 10.1186/s12902-019-0411-2 (PMC6683451; doi:10.1186/s12902-019-0411-2)
Supplement: Supplementary file 2 — Posterior probabilities associated with each profile in the six-profile model (N = 147). Posterior probabilities associated with each profile in the six-profile model (N = 147). (DOCX 16 kb) [file 12902_2019_411_MOESM2_ESM.docx]

Additional file 2. Posterior probabilities associated with each profile in the six-profile model (N=147).

| Profile | n (%) | Profile 1 | Profile 2 | Profile 3 | Profile 4 | Profile 5 | Profile 6 |
| --- | --- | --- | --- | --- | --- | --- | --- |
| 1 | 16 (10.9) | **0.935** | 0.020 | 0.005 | 0.025 | 0.016 | 0.000 |
| 2 | 14 (9.5) | 0.000 | **0.977** | 0.011 | 0.000 | 0.012 | 0.000 |
| 3 | 23 (15.6) | 0.001 | 0.016 | **0.962** | 0.021 | 0.000 | 0.000 |
| 4 | 29 (19.7) | 0.001 | 0.000 | 0.045 | **0.904** | 0.025 | 0.024 |
| 5 | 28 (19.0) | 0.000 | 0.030 | 0.000 | 0.005 | **0.963** | 0.002 |
| 6 | 37 (25.2) | 0.000 | 0.000 | 0.015 | 0.028 | 0.010 | **0.946** |
